# The Technological Condition of Human Evolution: Lithic Studies as Basic Science

**DOI:** 10.1007/s41982-021-00098-1

**Published:** 2021-08-27

**Authors:** Shumon Tobias Hussain, Marie Soressi

**Affiliations:** 1 Department of Archaeology and Heritage Studies, School of Culture and Society, Aarhus University, Moesgård Allé 20, 8270 Højbjerg, Denmark; 2 Faculty of Archaeology, Leiden University, Einsteinweg 2, 2333CC Leiden, The Netherlands

**Keywords:** Human origins, Paleolithic, Lithic technology, Homo faber, Archaeological sciences, Archaeological knowledge

## Abstract

The recent elaboration and rapid expansion of aDNA, paleoproteomics, and related fields have propelled a profound “biomolecular turn” in archaeology and fundamentally changed the topology of archaeological knowledge production. Such a transformation of the archaeological research landscape is not without consequence for long-standing research practices in the field, such as lithic analysis. This special issue derives from the session *Old Stones, New Eyes?* organized by the authors at the UISPP World Congress in Paris in 2018, which aimed to explore the future of lithic studies. An underlying theme of our session was the felt need to respond to the increasing marginalization of lithic research in terms of its capacity to (1) contribute to the grand narratives of early human evolution and (2) better articulate the role and significance of lithic studies in interdisciplinary human origins research. In this editorial, we briefly outline some of the questions and challenges raised by the biomolecular turn and advocate for a more self-conscious and reflexive stance among lithic experts. We argue that lithic studies fulfill all necessary requirements to act as a basic science for human origins research and that its role and status depends less on technological advances, such as, e.g., improved computing facilities, novel analytical software, or automated shape capture technologies, than on continuous work on the conceptual and methodological foundations of inquiry. We finally draw attention to the unique capability of lithic studies to shed light on the human technological condition and illustrate this potential by introducing and briefly discussing the papers included in this issue.

## After the “Biomolecular” Turn: Questions and Challenges for Lithic Research

Archaeology has changed dramatically over the last decades and has witnessed the development and diversification of independent branches of knowledge production within the archaeological sciences and “biomolecular” research (Bösl, 2017; Brown & Brown, 1992, 2011; Brown & Brown, 2013; Cappellini et al., 2018; Jones, 2002; Krause, 2019; Reich, 2019). Seen by many as “revolutionizing” our knowledge of the past (Evershed, 2008; Renfrew, 2010), the "biomolecular turn" has added a whole new dimension of data and insight to the accruing picture of early human evolution. The crystallization and spectacular success of biomolecular archaeologies has been a true game changer for broader human origins studies (cf. Goodrum, 2009; Schroeder, 2020). Some of these fields have seen a radical re-organization of knowledge-building resources and institutional infrastructures and thus a latent transformation of their guiding logic of research, catalyzed by the biomolecular boom and the growing significance and authority of the archaeological sciences both in the academic world and in wider society.

The study of ancient DNA, ancient proteins, or various biomarkers are now widely identified as “cutting-edge” divisions at the forefront of the field where new discoveries are made at an astonishing pace, methodological innovation is ubiquitous, and the most substantial methodological advances and breakthroughs are expected, ultimately resulting in a near-complete overhaul of how we understand and frame human beginnings. Symptomatically, however, many practitioners feel that at the same time biomolecular approaches, flanked by other “scientific” endeavors in paleoarchaeology such as isotope studies, have begun to assault the terrain of traditional archaeological research, in many cases implicitly or explicitly aspiring to take the lead and to set new agendas in the study of past population histories, demography, mobility, social networks, environments, and subsistence practices, to name just a few of the concerned research areas. aDNA in particular is thought to finally provide answers to some of the long-standing key questions of our species’ evolutionary history which traditional approaches have wrestled with almost since the inception of archaeology as an academic discipline (cf. Reich, 2019; Renfrew, 2010). While the promise of the ongoing biomolecular turn for refining and broadening our apprehension of human evolution is undeniably colossal, it therefore simultaneously forces us to re-consider what role remains for other long-established research practices in the field which often provide at first glance less spectacular results, such as stone artifact analysis. What is the contribution of lithic studies to the bigger picture of human origins? Do lithic scholars have to re-imagine their contribution and chart new ground to remain relevant? Or has the vital contribution of stone artifact research rather been forgotten, overlooked, and/or unjustifiably marginalized?

We pose this question not because we concede to those who proclaim that lithic studies have little new or exciting to say—to the contrary. Yet, we believe it is perhaps as important as never to reflect on the “added value” of the lithic research endeavor in the increasingly interdisciplinary dance of fields and specializations now interrogating the human deep past and the increasingly relevant question of what makes us human. It is arguably no longer enough to assert that stones simply form the backbone of the Paleolithic archaeological record and that their documentation, analysis, and comparison are significant due to this circumstance alone. The question is whether the specifics and details of lithic knowledge production make a difference for how the human story is portrayed, framed, and narrated, for how paleoarchaeological discourses move forward as well as what questions are considered critical, urgent and imperative. The issue is also whether new discoveries, advancements, ideas, and theories promoted through lithic research have a real bearing on the nature, direction, and dynamics of broader human origins research, beyond the provision of baseline dates—for example, for the oldest stone tools—or for the establishment of generalized taxonomic and chronocultural frameworks.

We believe that there is at least enough doubt and ambiguity in answering these questions to warrant a critical conversation on the place, status, and input of lithic studies within the rapidly changing and differentiating landscape of human origins research. At the very least, there is a double obligation with which lithic practitioners find themselves increasingly confronted: (1) clarifying their vision, ambition, and mission in contributing to the cross-disciplinary investigation of early human evolution and (2) drawing attention to the indispensability of their input and to communicate the latter clearly and effectively to the wider research community. The issue here is not merely to render lithic research a more visible field of scholarly practice but to make room for new arguments and incentives—including the rejuvenation of old ideas—for building interdisciplinary bridges and collaborations that exploit the full potential of accumulating lithic knowledge and expertise, and articulate it better with the emerging knowledge ecologies of twenty-first century human origins research.

## A Basic Science Proposal

What has perhaps been overlooked so far, and to some extent repressed, is the fact that lithic studies disclose a unique field of investigation to map and understand the evolution of the human “technological condition” (sensu Stiegler, 1994, 2003), i.e., how different hominin forms engaged with increasingly object-enriched environments and developed technically mediated lifeways that would in turn shape the course of their millennial-scale biocultural evolution (cf. Chazan, 2019; Coward, 2016; Hussain & Will, 2020; Kelly, 2016). Examining and understanding this technological condition across deep prehistory—the *Homo faber* predicament of human evolution— may provoke a fundamental re-consideration of the potential role and significance of lithic studies in broader human origins research. Rather than grappling with the thorny, and most likely misleading, question of whether the stone tool evidence from the late Pliocene and Pleistocene informs us about social and cultural vectors of hominin life or instead merely reflects basic adaptive and ecological needs, putting emphasis instead on both the constraints and blessings of living in artificial worlds fosters the examination of recursive human-technology interactions and their long-term consequences for the dynamics of human evolution. Importantly, this formulation naturally draws attention to the inescapable status of lithic studies as a basic science insofar as being and becoming human comes then into view as a technologically mediated process starting at least 3 to 4 million years ago.

A basic science in the here-envisioned sense supplies the (1) empirical, (2) categorical, and (3) philosophical preconditions for a larger field of knowledge production (cf. Polyani, 1962; Godin, 2003; Calvert, 2006; Pielke, 2012; Schauz, 2014)—in the present case human origins studies. From an (1) empirical standpoint, technical objects, artifacts, and technological systems do not only represent the most robust and widespread source of evidence on the deep prehistory of the hominin lineage, they also enable the qualification and comparison of a whole range of key dimensions of hominin behavior, such as subsistence (Henry, 1995; Kuhn, 1993), resource exploitation (Geneste, 1988, 1991; Kuhn, 2014), mobility (Kelly, 1988; Binford, 1980; Nelson, 1991), social transmission and interaction (Klaric, 2018; Pigeot, 1986; Tostevin, 2019), cognition (Gamble et al., 2014; Gowlett, 1984; Pelegrin, 2005; Pigeot, 2011; Stout et al., 2014), cultural norms and logics (Bodu, 1994; Perlès, 1991; Slimak, 2019; Valentin, 2008), and adaptation (Bleed, 1986; Hopkinson, 2004; Torrence, 1989)—to many of which lithic studies promise to offer privileged or at least original access.

## Lithic Research as a Window into the Human Technological Condition

Following Stiegler (1994) and other theorists of technology, lithic studies permit us to ask and chart how the human condition was shaped by technical realities (material artifacts, ways of doing, knowledge, technological concepts, etc.) over many thousands and millions of years, and how hominin-world relationships were regulated by a range of extra-somatic artifacts and technologies. These technical realities may then not only be regarded as a repertoire of “instruments” to work upon encountered natural surroundings, extract resources and/energy from the environment, or enact “adaptive interfaces” (Churchill, 2014; Isaac, 1977; Kuhn, 1993) but may instead come into view as co-constituting the basic conditions of hominin life itself—promoting unique possibilities of action and evolution and changing the logics and rationalities of navigating the various environmental, artifactual, social, and cognitive worlds past hominins inhabited (Chazan, 2019; Hussain, 2018; Hussain & Will, 2020; Pesesse, 2018). The production and use of stone tools emanate in this way as central evolutionary factors, co-regulating long-term dynamics of change and re-configuring selective pressures (cf. Iovita et al., 2021). In his seminal treatment of the *faber* condition of hominin evolution, Sigaut (2012: 7, 132, 187) sought to capture this processual co-adjustment of hominins and their stone tool environments by emphasizing that hominin behavior is always already “outfitted action” (*action outillé*), thus fundamentally altering how hominins intersect with their surrounding world and promoting “shared” and/or “sponsored” foci of attention and experience (*attention partagé*, *partage de l’expérience*) within an ever-evolving human-technology nexus. Technologically mediated actions and behaviors, in this view, do not simply come after perception, but rather the other way around: technologies and artifacts can fundamentally steer the perceptual and problem-solving compass of their users, and what is perceived is then precisely what is of interest for technical action (cf. Guchet, 2018).

From this perspective, technical mediation is arguably a central facet of the “human niche” (Fuentes, 2017) and even though there is considerable debate on the centrality and autonomy of different lithic practices in the past, the overall abundance of stone artifacts and knapping debris and the often sophisticated nature of stone working technologies already early on (Delagnes & Roche, 2005; Roche & Texier, 1991) make it generally difficult to question the basic value of lithic studies for understanding the evolutionary background of our species. Already the lithic worlds that emerged in the Oldowan clearly exceed the material environments of extant nonhuman primates by several orders of magnitude (Toth & Schick, 2009), and it seems clear that these increasingly furnished and object-enriched worlds crucially mediated evolutionary processes within the hominin lineage (cf. Shea 2017; Kuhn 2021; Stout 2021). The key point is that stone artifacts are much more fundamental to human evolution than is often acknowledged, and the changing role of these artifacts and their technological context through time forms an irreducible knowledge concern in wider human origins research.

From a (2) categorical point of view, the arguments for the basic science status of lithic studies are even stronger since a large suite of baseline terminology and essential research concepts both commonly and widely employed in human origins research directly derive from, or at least speak to, the lithic record (cf. Reynolds & Riede, 2019). The classic Clarkian sequencing of the deep past into “Mode 1–5” lithic industries (Clark, 1969)—recently disaggregated and refined by Shea (2013)—is but one example of a fundamental set of categories which provides structure to how we handle and represent the hominin past, and continues to guide empirical investigations, interpretations, theories, and meta-narratives of hominin evolution (Kuhn, 2021). In a similar vein, terms such as “Acheulean,” “Mousterian,” “Aurignacian,” or “Magdalenian” are routinely deployed by archaeologists and non-archaeologists alike to frame and organize this past (Boissinot, 2015), and these terms are deeply entangled not only with different epistemological assumptions but also with varying sets of observations and inferred patterns of lithic variability (cf. Hussain, 2019; Roebroeks & Corbey, 2001).

Basic, and often bipolar, categorizations of Paleolithic technological practice such as *débitage* vs. *façonnage*, various kinds of prepared core technology and alternative modes of volume management (e.g., “surface” vs. “volume” exploitation; cf. Boëda, 1994) as well as different kinds action chains (e.g., “hierarchical” vs. “non-hierarchical”) or stylized reduction methods such as “Levallois,” “Discoid,” or “Quina” anchor our understanding of hominin cognitive evolution (Abramiuk, 2012; de Beaune et al., 2009; Lombard et al., 2019; Pelegrin & Roche, 2017), and thus scaffold how we reconstruct and assess the deep history of human technology (Kuhn, 2021; Tomlinson, 2018), the evolution of hominin motor skills (Key et al., 2020; Stout, 2011; Stout et al., 2008), or the presumable uptake of hominin cultural capacities (Haidle et al., 2011, 2015). The stone tool evidence is indeed so central to framing our imaginaries and explanations of the hominin past that many of the mentioned categories and named archaeological units have recently come under pressure (Bar-Yosef & Van Peer, 2009; Monnier & Missal, 2014; Riede et al., 2019; Shea, 2014; Wilkins, 2020), and criticism about the all-too-common “fetishization” and “reification” of these categories is now increasingly voiced from many different directions (Dibble et al., 2017; Reynolds & Riede, 2019; Tostevin, 2011). Independently of whether we agree with these critiques and the arguments they mobilize or not, the polarizing discussion and its controversies at the very least illustrate the epistemic gravity of basic research categories derived from the stone artifact record. How we construct, frame, and use these categories often makes a crucial difference for how the past comes into view and can be analyzed, and how multidisciplinary research cuts the human story “at its joints.”

Historically, lithic studies have also played a major role in establishing the (3) philosophical preconditions of knowledge production in human origins research. Although paleoarchaeologists, perhaps in contrast to other specializations in the field, have arguably long operated in a broader space of interdisciplinarity, stone artifact inquiry has actively shaped the development and elaboration of a core canon of research concepts and epistemologies of its own. Again, this contribution becomes especially lucid when the great controversies and most divisionary knowledge conflicts in the field are taken into account (Anghelinu et al., 2020; De la Torre & Mora, 2009; Hussain, 2019; Perlès, 2016; Tostevin, 2011). An instructive example is the continuing tension between lithic research based on *chaîne opératoire* theory and epistemology and approaches trending towards mechanistic reduction theories, including some readings of “the reduction thesis” (Jelinek, 1976; McPherron, 1994; Dibble et al., 2017: 827; cf. Hussain, 2019). The important point here is that these clashing approaches do not merely signal the adoption of different and frequently incompatible super-theories and research epistemologies. The ongoing engagement with the lithic evidence and its specific challenges of knowledge production has over the course of disciplinary history led to the development of different benchmark premises and assumptions, but also resulted in the internalization of specific categories of inquiry, interpretive preferences, and the cultivation of divergent understandings of fundamental concepts such as “technology,” “assemblage,” and even of what a stone “tool” is (cf. Hussain, 2019).

The status of lithic studies as a basic science with the capacity to stipulate conceptual change and innovation is further illustrated by ongoing attempts to re-think taken-for-granted research categories, for example, in relation to the concept of the lithic tool (cf. Forestier & Boëda, 2018), to develop new ways of understanding and analyzing social learning processes (Leroyer, 2016; Mesoudi and Aoki, 2015; Tostevin, 2019), or to re-map long-term constellations of technological diversity, complexity, and cutting-edge efficiency (Kuhn, 2021; Muller et al., 2017; Perreault, 2019; Režek et al., 2018; Stout et al., 2010). Another important illustration is the transfer of central concepts and approaches from lithic analysis to other domains of paleoarchaeological inquiry. An obvious example is the implementation of *chaîne opératoire* principles to the study of mobile and parietal art (Farbstein, 2011; Lorblanchet, 1994, 2010; Tosello & Fritz, 2004). Similar processes can be recognized for the analysis of tool designs and object biographies with regard to organic tools and technologies, which is at least in part modeled on lithic research (Julien, 1992; Langley, 2016). The study of ceramic technologies yields yet another example of such methodological translation (see esp. Roux, 2016). The transfer of key concepts and analytical approaches demonstrates that lithic studies continue to frame larger knowledge production processes and have the capacity to supply some of the philosophical preconditions for different strands of inquiry in human origins. And even though this agenda-setting capability of lithic studies is not always exploited or recognized as such, for example in the context of paleoarchaeological research programs that emphatically model themselves on the “hard sciences,” we would argue that it is a missed opportunity at best to borrow by default from other disciplines without also considering, and actively exploring, the possibilities in which concepts and insights developed in response to the issues raised by the lithic evidence can benefit and shape other (neighboring) disciplines, and in this way re-configure broader knowledge concerns in human origins.

While the biomolecular turn may challenge the exclusive agenda-setting prospects of lithic research in human origins studies, it remains to be seen whether biomolecular research can itself be developed into a basic science in the above-specified sense. We should not lose sight of the fact here that the biomolecular turn and the applause that it currently receives is largely fueled by technological advances. From the perspective of basic science concerns, however, it is a virtue rather than a burden to produce comparatively “unspectacular” findings and to recede from result-oriented research interests and quick dissemination strategies. Basic science, in this view, at least includes a “slow” component of knowledge production in which the evidence is continuously expanded, archived, reviewed, re-analyzed, and re-conceptualized and in which “data” is considered as important as reflecting upon the preconditions of knowledge production. Basic science entails at least some knowledge "banality".

In total, it may thus be argued that the perceived difficulties of lithic scholarship to carve its proper place in the study of early human evolution, to counter the threat of marginalization, and to dismantle what might be referred to as a “lithic inferiority complex” among lithic scholars are in part a product of the lack of self-recognition as a basic science. As such basic science, lithic studies need to continue struggling with the fundamental questions of its knowledge domain, invest in and defend “slow” and time-consuming, and often transgenerational, fieldwork and research, and seek to continuously frame and reframe questions, concepts, and knowledge ecologies with the potential to alter and transform how we grapple with the remote human past. Lithic scholars need to be more transparent about the strengths and weaknesses of their evidence but also about their constructive and critical knowledge competences, and they should begin to reflect more actively about their role in wider human origins studies, larger human evolutionary studies or even “big history” as a whole. What are the questions that only lithic scholarship can answer? And what are the facets of hominin evolution that only lithic experts have access to?

## This Special Issue: Sampling New and Re-imagining Old Perspectives

The papers gathered in this volume are the product of a concerted effort to promote new impulses in lithic paleoarchaeology as well as to map out and strengthen its contribution to inclusive understandings of early human evolution. The papers are revised versions of original contributions to a conference panel on new directions in lithic studies co-hosted by the authors at the UISPP World Congress 2018 in Paris, France. The goal and ambition of these papers are not so much to enlist a comprehensive body of timely considerations and perspectives, but rather to take some initial steps in re-situating and re-imagininge lithic inquiry in human origins studies and to demonstrate the lasting significance of stone artifact analysis as an independent critical knowledge domain. The first three papers gathered here deal with various forms and scales of hominin mobility and show that the lithic evidence is indispensable for understanding how early humans utilized and managed space and eventually transformed themselves into a global species. The last paper draws attention to the social dimensions of lithic technology and, in Paleolithic research, the still underdeveloped opportunities for theorizing and exploring sociotechnical dynamics in their own right. These four contributions identify different priorities, challenges, and advantages of advancing knowledge of the deep past through lithic research but they all make important observations and table weighty arguments for why lithic studies should retain their proper place at the heart, and in some instances arguably at the helm, of human origins studies.

Kuhn (2020) argues that mobility is a derived behavioral characteristic within the hominin lineage and a key feature of the human condition. He contends that this *Homo movens* predicament can primarily be accessed and qualified by mobilizing the broad sweep of the available lithic evidence. “Mobility thinking,” which has a long history in archaeology and today belongs to the core lexicon of the discipline, must be updated in light of new and emerging research of broad, cross-disciplinary concern and this could be a potent way of re-inserting lithic studies into key human origins discourses. Kuhn identifies and outlines two principal opportunities of mobility-oriented lithic research. The first is to link the findings and patterns derived from lithic mobility studies to major developments in the evolution of hominin biology. He argues that mobility provides the “essential link between anatomy and ecology,” and we should therefore develop more systematic and focused approaches to investigate how variation within the two intersects through space and time. Kuhn suggests that it is indeed time to map out large-scale patterns of mobility and relate them to changes in hominin body plan, locomotor efficiency, mode of bipedality, dispersal capacity, and diet. The second opportunity is to develop a comparative research framework where data on raw material acquisition and provisioning are integrated and charted over extended spatial and temporal scales. Mobility is here not understood as a generic, somewhat fixed category, but rather as a flexible behavioral domain exhibiting meaningful variability on almost all scales of observation and analysis.

Kuhn (2020) identifies three promising domains of inquiry that may help to reconnect lithic mobility thinking to larger concerns in paleoanthropology and human evolutionary studies. The first is the re-assessment and comparison of hominin territorial geometries (sizes and configurations of territories) and ranging patterns. The second theme is the study of Paleolithic social networks and their millenial-scale evolution. Given that social networks structure ecological and evolutionary dynamics and play key roles in cultural evolution by influencing social transmission patterns and rates of change, lithic specialists must begin to pay more attention to the differential movement and circulation of different kinds of stone artifacts including the modes and conditions of transportation and mobility-mediated raw material transformation trajectories (re-sharpening, life history, etc.). Thirdly, lithic studies should be joined with larger research efforts in social evolution in order to effectively investigate questions of cooperation and “activity differentiation” within hominin groups or populations. Kuhn rightly points out that lithic mobility research has too often assumed fairly homogeneous, hypothetical groups as the central units of past mobility. But it is perhaps the differentiation, individuation, and diversification of mobility choices, requirements, strategies, and regimes within larger social formations that provide meaningful evolutionary perspectives on the lithic archaeological record. Kuhn offers a host of concrete and easily operationalizable considerations for how to propel these three areas of lithic investigation forward and correctly stresses that what is mostly required here has less to do with technical and methodological advancements but instead demands a careful (re-)adjustment of research questions and conceptual premises.

Holdaway and Davies’ contribution (2020) seeks to reinvigorate the study of lithic surface assemblages for understanding landscape-scale variability in human evolution. They similarly call for a fundamental re-consideration of guiding assumptions and theoretical convictions in the analysis of human mobility systems and settlement dynamics. Holdaway and Davies criticize center-periphery models that often implicitly underpin landscape archaeological research or settlement pattern analysis in prehistoric archaeology. The authors argue that the arbitrary dichotomization between settlement and activity nodes and empty spaces in-between them does in practice little justice to the complexity and often continuous nature of lithic landscapes. Holdaway and Davies tie this critique to a focused review of anthropological debates on hunter-gatherer bands and their relationships with the land. They show that forger spatial organization is typically highly dynamic and amendable to change even within the same annual cycle. Mobile forager societies rarely exhibit fixed or stable spatial correlates. Their land-use and tenure practices are continuously negotiated on different social and organizational levels, including the context-dependent aggregation and mixing of people and emergent, situational activity profiles of individuals. Similar to Kuhn (2020), they argue that lithic studies that target landscape-scale patterns of artifact distribution and composition should refrain from the assumption that the responsible agent was a bound, homogeneous group or population.

Holdaway and Davies (2020) therefore suggest that a fundamental shift in how we conceive of the archaeological surface record and its formational dynamics is required in order to clear the ground for new insights on the variability of spatial behavior and diversified forms of spatial organization in human evolution. Rather than regarding landscape-scale patterns in lithic artifacts as reflections of specific tasks carried out at particular places or of larger settlement systems with complementary types of sites that can be unequivocally located in time, these patterns should be recognized as emergent outcomes of time-averaged surface collections, recording the continuous transport, discard and re-working of stone on different spatial scales and with often varying temporalities. The authors draw on the metaphor of the “field” to shift the attention away from static systems and patterns to the importance of raw material flows themselves, and they underscore the need to pay more attention to the emergent potentials of an accumulating yet constantly changing surface record.

Drawing on simulation results and an archaeological case study from Holocene semi-arid Australia, Holdaway and Davies (2020) argue that what is missing from surface locations is often more informative than what is present, and that the composition of lithic assemblages at particular places—in stark contrast to received wisdom in the field—therefore primarily informs about human behavior elsewhere. This not only undermines presuppositions on the existence of strongly structured relationships between human sociocultural organization, mobility strategies, land-use and tenure and observed patterns in the lithic record, but also opens up the possibility for developing alternative research approaches to landscape-scale artifact patterns grounded in formation theory and some branches of complexity thinking. Holdaway and Davies invoke the example of Rutherfords Creek to demonstrate that a geometric approach based on the calculation of cortex ratios can provide a first simple means to chart flow dynamics of lithic fields in the landscape and to compare them through time and space. Their theory-driven perspective puts strong emphasis on the generative character of lithic landscapes and might be seen as a “third way” of practicing landscape archaeology, alongside traditional behavioral-ecological and technoanthropological approaches.

Zwyns (2021) returns to the issue of hominin range expansions, re-visiting the thorny question of how the lithic record might be used to better map and understand planetary-scale dispersal on evolutionary timescales. Although it is clear that the epithet of *Homo migrans* describes all but a peripheral facet of being and becoming human, there is much controversy and skepticism when the archaeological record is mobilized in order to chart and detail the deep history of human migrations. Zwyns argues that it is important to carefully think about what features and qualities of the material record are most likely to convey relevant information about large-scale hominin dispersal dynamics, and that it is thereby crucial to avoid the simple equalization of all available data. He rightly suggests that the quality and not the quantity of lithic information is key here. Zwyns maintains that in order to confidently identify and understand the lithic signatures of dispersal and intercontinental movement, we must first understand how widespread eligible lithic phenomena are and how likely observed changes are to occur in situ. We must, in other words, better understand what features of the lithic record are most susceptible to change, which are probably most strongly constrained through their social and natural environments, how these features were likely transmitted through time and space, and what the observed rate of change in the varying lithic domains of concern is. Zwyns contends that technological conceptions and core-blank systems are in many cases the privileged sources for reconstructing dispersal processes—they tend to be transmitted in packages rather than in sets of traits and their individual features and/or components can typically not be changed independently of other system components. Taking these systemic interdependencies into account presents a research opportunity rather than a burden.

Zwyns (2021) draws on the example of the Initial Upper Paleolithic (IUP) of central and eastern Asia to illustrate how such a technological approach to large-scale hominin displacements can be operationalized and broken down into analytical categories that enable the required scope of superregional comparison. The author proposes to initially focus on two vectors of lithic analysis in order to build an empirical baseline for identifying and understanding hominin dispersal dynamics: ubiquity (broken down into a regional and chronological sub-axis) and complexity. Ubiquity assessments, coupled with observation on patterns of variability and rates of change, allow a determination of the novelty of the recorded technological configurations, while complexity measures indicate how likely these are to be explained in terms of technological convergence.

Zwyns (2021) demonstrates that the IUP of central and eastern Asia is regionally but not chronologically ubiquitous enough to represent a serious dispersal candidate. IUP blade technology is further shown to be relatively complex when compared to other Middle Stone Age (MSA), Middle Paleolithic, or Upper Paleolithic core reduction technologies. Together with the observation that the larger blade production and transformation structure of the IUP is comparatively stable and coherent, whereas lithic tool designs and toolkit compositions exhibit much more local and even site-specific variation, the evidence is thus taken to support an incoming, non-autochthonous technological package, most likely introduced by incipient *Homo sapiens* populations.

Högberg and Lombard (2020) take up a social constructivist approach and adopt what they call a “socio-technical framework” perspective to re-connect lithic studies with broader concerns in social theory. They argue that the evolution of technology is not only powered by technology-specific properties, ecological qualities such as performance, efficiency, and cost–benefit trade-offs, or the innovative potential of individuals and populations, but larger patterns of stability and change are instead at least partly controlled by the wider sociological context in which specific technologies occur—by historically situated and context-specific societal norms, attitudes, desires, and beliefs. The authors propose that the life cycle of larger technological regimes thus opens a window into the formative dynamics and interactions at the technology-society interface, and that different stages of technological development—discovery, adoption, growth, stabilization, and demise—inform us about the configuration of associated social milieus, in particular in terms of social transmission processes. They derive their core arguments from Science and Technology Studies (STS), the sociology and history of technology, and the comparative study of socio-technical systems and illustrate the benefits of stage-theoretical explanations of coupled socio-technical change by discussing the modern examples of the bicycle, pneumatic tire, and electric guitar. Drawing on the seminal work of Dutch theoretician Wiebe Bijker, Högberg and Lombard contend that the implementation and eventual integration of technology into human society inescapably take time, and that the consecutive stabilization and systematization of specific technological practices are necessary requirements for their material correlates to persist in the archaeological record. Central to their approach is Bijker’s notion of “interpretive flexibility,” counteracting the idea that technological change is fixed on a given trajectory or that the in-built potential and utility of technologies, once adopted, largely determine the course of social evolution.

Högberg and Lombard (2020) muster two case studies—ethnohistoric Kimberly point production technology of northwestern Australia and MSA Still Bay point-making technologies from southern Africa—to assess the benefits of the socio-technical framework perspective in understanding the remote past. They show that Kimberley point production went through a series of technological phases where the goals, norms, sophistication, and cultural transmission context of technical practices changed in response to altered societal conditions, most notably the increasing interaction with European settlers which fundamentally transformed the traditional belief and prestige systems surrounding raw material acquisition and point manufacture. Ultimately, these remodeled sociocultural conditions destabilized evolved ways of making Kimberley points, systems of knowledge transfer and associated exchange networks and consequently changed the status of the points themselves, resulting in the eventual disappearance of Kimberley points from the material record. In the case of South African MSA Still Bay points, the authors suggest that large-scale geographical patterns in typo-technological variability can similarly be explained by changing cultural transmission contexts and the long-term life cycle of Still Bay point technology. By integrating aDNA-derived demographic estimates, luminescence dating evidence, paleoenvironmental information, and data on patterns of variability in Still Bay point technotypology, Högberg and Lombard illustrate how the MSA Still Bay point phenomenon emerged out of a pre-Still Bay context of flexible technological practice and became institutionalized before the onset of MIS 4 when effective population size appears to have declined and African-wide climate conditions ameliorated. The authors elucidate the shifting interplay between stable macro-regional patterns in point production and morphometry and regionally variable practices and design concepts. They argue that these dynamics can ultimately be understood as expressions of re-organizing networks of social interaction and knowledge transfer and the changing status and social significance of Still Bay points through time.

Taken together, the contributions to this special issue demonstrate that lithic studies have great potential to contribute to larger concerns in human evolutionary studies, advancing core areas of knowledge production and inspiring but also re-configuring our narratives of the deep past. They also show that the role of lithic analysis in the multidisciplinary study of human origins is bound to our capacity to re-imagine lithic research and to continuously work on its intellectual foundations. Lithic studies, for this reason, should strive for more epistemological, theoretical, and conceptual engagement with its empirical source material, not less. This ambition also calls for tailoring theories and concepts to the specificities of the lithic record, rather than simply transferring them from other domains of inquiry. The set of papers in this volume undoubtedly showcases the unshaken proclivity of lithic studies to act as a basic science for human evolutionary studies and illustrate that progress in lithic knowledge production does not only depend on technological breakthroughs or cutting-edge analytical techniques. What is required to re-insert lithic studies to the heart of human origins research is also a more careful consideration of what lithic research can offer, what concepts and theoretical perspectives are needed to strengthen and expand existing lines of inquiry, and what lithic scholars can do to open up novel areas of prospective insight and interdisciplinary debate.
